# Supplementary material for: Room-temperature quantum nanoplasmonic coherent perfect absorption
Source: Nat Commun. 2024 Jul 27;15:6324. doi: 10.1038/s41467-024-50574-9 (PMC11282272; doi:10.1038/s41467-024-50574-9)
Supplement: Supplementary file 1 — Supplementary Information [file 41467_2024_50574_MOESM1_ESM.pdf]

Supplementary Information for Room-temperature Quantum Nanoplasmonic Coherent Perfect Absorption

Yiming Lai[1,+], **Daniel D. A. Clarke**[1,+], **Philipp Grimm**[2], **Asha Devi**[1], **Daniel Wigger**[1], **Tobias Helbig**[3], **Tobias Hofmann**[3], **Ronny Thomale**[3], **Jer-Shing Huang**[4,5,6,7], **Bert Hecht**[2,>], **and Ortwin Hess**[1,*]

[1]School of Physics and CRANN Institute, Trinity College Dublin, Dublin 2, Ireland
[2]Nano-Optics & Biophotonics Group, Department of Experimental Physics 5, and Röntgen Research Center for Complex Material Research, Physics Institute, University of Würzburg, Am Hubland, 97074 Würzburg, Germany
[3]Theoretische Physik I, Julius-Maximilians-Universität Würzburg, Am Hubland, 97074 Würzburg, Germany
[4]Leibniz Institute of Photonic Technology, Albert-Einstein Strasse 9, 07745 Jena, Germany
[5]Institute of Physical Chemistry and Abbe Center of Photonics, Friedrich-Schiller-Universität Jena, Helmholtzweg 4, D-07743 Jena, Germany
[6]Research Center for Applied Sciences, Academia Sinica, 128 Sec. 2, Academia Road, Nankang District, Taipei 11529, Taiwan
[7]Department of Electrophysics, National Chiao Tung University, Hsinchu 30010, Taiwan
[+]These authors contributed equally.
[>]hecht@physik.uni-wuerzburg.de
[*]ortwin.hess@tcd.ie

Contents

Supplementary Note 1: Scattering matrix and generalized coherent perfect absorption
Supplementary Note 2: Transfer matrix for the waveguide-dimer structure
Supplementary Note 3: Nanowire eigenmode dispersion analysis
Supplementary Note 4: Nanocavity eigenmodes under gCPA
Supplementary Note 5: Electrodynamic simulations
Supplementary Note 6: Maxwell-Bloch simulations
Supplementary Note 7: cQED model of photoluminescence
Supplementary Note 8: Quantum steady-state analysis of a continuously pumped Jaynes-Cummings system
Supplementary Note 9: Movies of the nanocavity-emitter system under qnCPA

Supplementary Note 1. Scattering matrix and generalized coherent perfect absorption

Coherent perfect absorption (CPA), characterized by the ideal absorption of light at selected frequencies, is a rather special condition that can occur in systems which scatter light and exhibit lossy resonances[1,2]. It arises from a delicate interplay between the interference and dissipation of wave fields, such as to ensure a complete suppression of all outgoing radiation despite finite-amplitude incoming waves. In conventional dielectric photonic systems, one is frequently concerned with a finite number of input and output modes, where for example, light is coupled between waveguides and lossy cavities. In such a scenario, the primary dissipation mechanism is heat generation (i.e., non-radiative), and a scattering matrix (S-matrix) comprising the coupling coefficients between the incoming and outgoing optical modes is entirely adequate to capture the modal conversion and energy exchange within and between devices[3]. The condition for CPA is then strictly tantamount to the vanishing of an eigenvalue of this total S-matrix, and the corresponding CPA eigenmode represents a solution of Maxwell's equations that satisfies purely incoming-wave boundary conditions[2]. In contrast to dielectric resonators however, plasmonic ones offer much more flexibility, since they support bright as well as dark modes whose properties can be synthetically tailored. The absorbed energy can thus be converted to heat or even radiated, where the latter possibility is largely neglected in the original concept of CPA.

In a previous publication[4], we extended the traditional notion of CPA appropriately for plasmonic resonators that feature both non-radiative and radiative loss channels. Therein, we established that the concept of CPA, which requires at least one eigenvalue of the global S-matrix to vanish, can be generalized to scenarios in which this applies only to a particular sub-matrix. In particular, by finding and zeroing a chosen eigenvalue of the scattering sub-matrix appropriate to a single, guided input mode of a plasmonic waveguide, it was shown that ideal impedance matching (i.e., reflectionless energy transfer) with a nearby nanoantenna could be achieved in either its sub-radiant or radiant modes, thereby giving rise to an efficient source of heat or far-field radiation respectively. As such, this generalized CPA (gCPA) is a useful conceptual tool that makes the notion of perfect absorption meaningful in situations where the behaviour of the guided modes alone is inadequate for a proper understanding; especially for plasmonic nanoresonators, it allows one to describe and quantify the selective absorption of a given input mode in the presence of (infinitely many) radiative loss channels, without the need to fulfill the requirements of CPA for the complete system. Our approach is formally equivalent to the generalized theory of reflectionless scattering modes recently proposed by Sweeney *et al.*[5,6], but was developed independently to address the coupling of a guided, subwavelength surface plasmon mode to an optical nanoantenna.

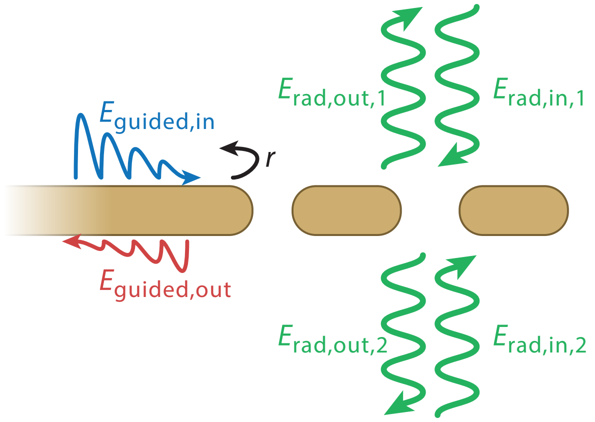

Supplementary Figure 1. Scattering channels in a nanoplasmonic waveguide-driven dimer nanocavity. Schematic of the nanowire-dimer system analyzed in the main text. In general, guided surface plasmon modes $\mathbf{E}_{\text{guided}}$ as well as radiative modes $\mathbf{E}_{\text{rad}}$ must be accounted for. The formalism of gCPA allows one to describe and quantify the absorption of a selected waveguide input mode in the presence of far-field radiation losses, which are treated as complementary dissipation channels.

We invoke this generalized picture of CPA here by treating radiation into the far-field as constituting additional loss channels, besides Ohmic dissipation. Supplementary Fig. 1 illustrates the input and output modal fields of the nanoplasmonic wire-dimer

system considered in the main text, which can be linked via the matrix relation

$$\begin{pmatrix} E_{\text{guided,out}} \\ E_{\text{rad,out,1}} \\ E_{\text{rad,out,2}} \\ E_{\text{rad,out,3}} \\ \vdots \end{pmatrix} = \mathbf{S}_{\text{global}} \begin{pmatrix} E_{\text{guided,in}} \\ E_{\text{rad,in,1}} \\ E_{\text{rad,in,2}} \\ E_{\text{rad,in,3}} \\ \vdots \end{pmatrix}.$$

Here, the global S-matrix, $\mathbf{S}_{\text{global}}$, connects all incoming with all outgoing modes and is defined by

$$\mathbf{S}_{\text{global}} = \begin{pmatrix} r & c_{g1} & c_{g2} & \ldots \\ c_{1g} & d_{11} & d_{12} & \ldots \\ c_{2g} & d_{21} & d_{22} & \ldots \\ \vdots & \vdots & \vdots & \ddots \end{pmatrix},$$

where r denotes the reflection coefficient of the guided surface plasmon mode of the nanowire (due to the hemispherical termination), c_{ij} are the modal coupling coefficients between the guided mode and radiative modes, and d_{ij} describe the coupling among radiative modes. For the particular system under study here, we provide feeding power only via the guided mode of the nanowire, (i.e., $E_{\text{rad,in},i} = 0$ for all i). The objective of our gCPA analysis is to find a zero eigenvalue for the non-zero input subspace. Technically, this means that the complex-valued reflection coefficient r must vanish, so that we achieve a unidirectional, near-field power coupling from the waveguide to the dimer nanocavity. At the gCPA condition, the remaining entries of the output vector will in general not be zero, tantamount to finite far-field radiation amplitudes.

Supplementary Note 2. Transfer matrix for the waveguide-dimer structure

In order to compute the complex reflection coefficient r, the plasmonic dimer nanocavity is modelled as a pair of coupled Fabry-Pérot resonators that support the fed-in surface plasmon mode of the nanowire[7,8]. Reflection and transmission events occurring at the gaps and the open end, as well as the plasmon propagation loss, must be carefully included to provide a full account of phase-sensitive interference effects. They are treated numerically by means of rigorous mode expansion calculations within the framework of finite-difference time- and frequency-domain techniques (see below). Using this information, the transfer matrix analysis discussed in Ref.[4], and schematized for our system of interest in Supplementary Fig. 2, provides an elegant approach to calculate reflection coefficients as a function of wavelength, rod lengths and gap sizes. In particular, it offers a transparent description of the gCPA mechanism, where the directly reflected wave at the nanowire termination (illustrated in green) exhibits equal amplitude, but opposite phase, relative to the coherent superposition of waves reflected back from the gold dimer onto the wire (illustrated in red), giving rise to complete destructive interference of outgoing modes.

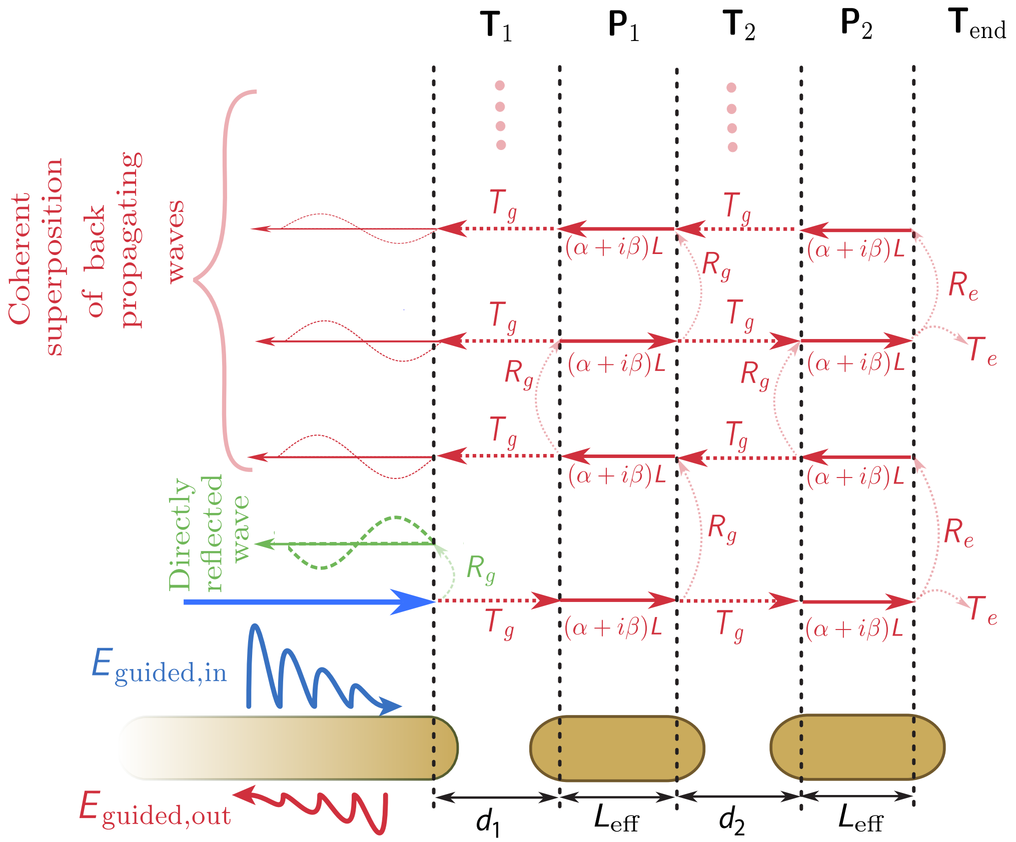

Supplementary Figure 2. Schematic representation of the interference effects and the transfer matrix analysis of a nanoplasmonic waveguide-driven dimer nanocavity. Visualization of the sequence of transfer matrices which describe the signal transmission behavior across an obstacle ($\mathbf{T}_i$) and the propagation behaviour along a regular guiding section ($\mathbf{P}_i$) throughout the nanoplasmonic wire-rod-rod structure.

The schematic provides a comprehensive visualization that incorporates the relevant reflection and transmission coefficients required to assemble the 2×2 gap transmission matrix $\mathbf{T}_i$ and the propagation matrix $\mathbf{P}_i$,

$$\mathbf{T}_i = \frac{1}{T_g}\begin{pmatrix} T_g{}^2 - R_g{}^2 & R_g \\ -R_g & 1 \end{pmatrix}, \; \mathbf{P}_i = \begin{pmatrix} e^{i(\beta+i\alpha)L_{\text{eff}}} & 0 \\ 0 & e^{-i(\beta+i\alpha)L_{\text{eff}}} \end{pmatrix}, \tag{1}$$

as well as the open-end matrix $\mathbf{T}_{\text{end}}$,

$$\mathbf{T}_{\text{end}} = \frac{1}{T_e}\begin{pmatrix} T_e{}^2 - R_e{}^2 & R_e \\ -R_e & 1 \end{pmatrix}. \tag{2}$$

Here, the reflection coefficient R_g and the transmission coefficient T_g for an obstacle are assessed between two semi-infinite nanowires, R_e and T_e represent the reflection and transmission coefficients for the open-end termination of a semi-infinite

nanowire and the guided plasmon characteristics are included via the propagation constant β and attenuation constant α, determined by numerical simulations as explained in Supplementary Note 3. It is worth noting that the quantity L_{eff} appearing in these formulae refers specifically to the length of the cylindrical portion of each nanorod. The total 2×2 transfer matrix $\mathbf{M}$ for the guided nanowire mode is then obtained by multiplication,

$$\mathbf{M} = \mathbf{T}_{\text{end}} \times \mathbf{P}_2 \times \mathbf{T}_2 \times \mathbf{P}_1 \times \mathbf{T}_1.$$

The reflection coefficient r in the particular scattering submatrix of interest can be calculated via the entries of $\mathbf{M}$,

$$r = \frac{E_{\text{guided,out}}}{E_{\text{guided,in}}} = -\frac{M_{21}}{M_{22}} = -R_g - \frac{{T_g}^2}{-R_g + \frac{e^{2(\alpha-i\beta)L_{\text{eff}}}}{-R_g + \frac{R_e {T_g}^2}{R_e R_g - e^{2(\alpha-i\beta)L_{\text{eff}}}}}}.$$

Supplementary Note 3. Nanowire eigenmode dispersion analysis

To analyze the dispersion and electromagnetic near-field characteristics of the fundamental TM_0 eigenmode of a plasmonic nanowire waveguide, we employ the finite-difference frequency-domain method[9] as implemented in Mode Solutions, Ansys Lumerical. For each frequency or wavelength, a pair of values (β, α) indicates the allowed combination of propagation constant β and attenuation constant α, from which we can deduce a relation $\alpha(\beta)$ that describes the TM_0 mode via a curve in the complex wavevector plane (by eliminating the explicit frequency dependence), as demonstrated below in Supplementary Note 4. Note that here and throughout this work, we employ experimental single-crystal data[10] for the dielectric function of the constituent gold material.

Supplementary Note 4. Nanocavity eigenmodes under gCPA

In order to ascertain and unambiguously confirm the emergence of the gCPA regime in a passive, feeding-wire-coupled nanocavity, we perform transfer matrix calculations as described above (see Supplementary Note 2), thereby obtaining the reflection coefficient r in the two-dimensional, complex wavevector plane (i.e., as a function of propagation constant β and attenuation constant α). We plot in Supplementary Fig. 3a the reflectance $|r|^2$ and in Supplementary Fig. 3b the reflection phase, Arg r where Arg denotes the argument, in the complex (β, α) plane, focusing on the case of the bonding dimer resonance. Cavity resonances generally appear as pairs of extremal reflectivity features, where zeros are located in the loss region $(\alpha > 0)$ and poles in the gain region $(\alpha < 0)$. We show the dispersion characteristic of the TM_0 guided mode via the solid black line, indicating the allowed combinations of α and β for it. Just above 800 nm, the reflectance displays a zero and a pole (Supplementary Fig. 3a), accompanied by corresponding phase singularities (Supplementary Fig. 3b). To achieve true gCPA for the bonding mode of the nanorods, the phase singularity at which zero reflectance occurs must reside on the dispersion curve of the TM_0 mode[11], as is indeed the case in Supplementary Fig. 3. It is also worth commenting on the phase singularity present in Supplementary Fig. 3b, corresponding to a reflectance pole and being therefore oppositely winded; here, the singularity is associated with a negative field decay constant α, implying amplification of the guided plasmonic mode and the theoretical possibility of surface plasmon amplification by stimulated emission in such a system[12–14].

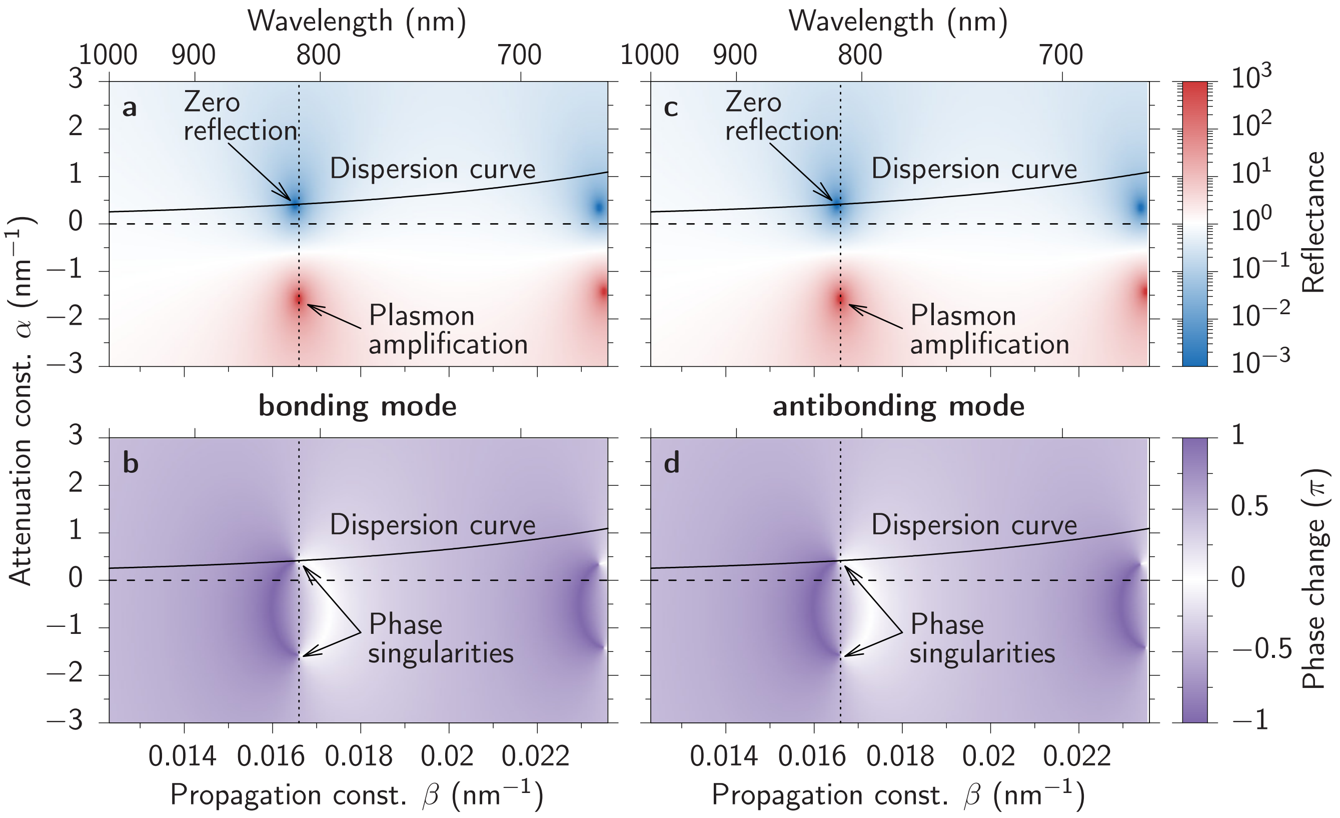

Supplementary Figure 3. Dimer nanocavity bonding and antibonding modes under gCPA. (a) Reflectance $|r|^2$ and (b) phase change upon reflection, Arg r, in the complex wavevector (β, α) plane for the bonding mode, calculated in accordance with the transfer matrix formalism. The reflection zero residing on the dispersion curve of the TM_0 guided nanowire mode (solid black line) corresponds to the bonding resonance of the dimer nanocavity, marking a gCPA state at a wavelength of just above 800 nm (indicated by the vertical dashed line). The phase singularity associated with the reflectance zero coincides with the dispersion curve at the same critical wavelength. The transfer matrix analysis yields optimal system geometrical parameters for gCPA in this case of $L = 114$ nm, $d_1 = 3.5$ nm and $d_2 = 3.0$ nm. These parameters are defined in Supplementary Fig. 2. (c) Reflectance and (d) phase change plotted in the complex wavevector (β, α) plane for the antibonding mode. The reflectance zero and pole, together with their corresponding phase singularities, are indicated. The optimal system geometrical parameters for gCPA in this case are $L = 166$ nm, $d_1 = 3.0$ nm and $d_2 = 3.0$ nm.

In addition to the bonding mode, we also demonstrate here that the antibonding mode can be driven under gCPA (i.e., ideal impedance matching) in a passive nanoplasmonic cavity-waveguide system. In order to meet the gCPA criterion, we shift the

reflection zero onto the dispersion characteristic of the TM_0 guided nanowire mode by systematically tuning L, d_1, and d_2 (parameters indicated in Supplementary Fig. 2). Supplementary Figure 3c depicts the resulting reflectance map, where the minimum associated with the antibonding cavity mode resides on the dispersion curve (black solid line) at a critical wavelength of 818 nm (see the figure caption for geometrical parameters). The associated reflection phase map is shown in Supplementary Fig. 3d, where the phase singularity that accompanies the reflection minimum is analogously found lying on the dispersion curve. Altogether, these results demonstrate that radiative as well as non-radiative nanocavity modes can be directly addressed by our proposed, near-field-mediated gCPA driving mechanism.

Supplementary Note 5. Electrodynamic simulations

In this study, we employ a combination of classical transfer matrix and full-dimensionality finite-difference time-domain (FDTD) simulations in order to systematically identify gCPA in a plasmonic waveguide-driven nanocavity system. The transfer matrix formalism has been described in previous work[4] and further details concerning our present application are given in the above sections. Our full-dimensionality FDTD simulations[15] of the spatiotemporal near-field dynamics are performed using Ansys Lumerical software. The TM_0 nanowire guided mode discussed above is directly employed as a source in our FDTD calculations. To ascertain the reflection characteristics of this mode, a two-dimensional, frequency-domain field monitor (oriented perpendicular to the nanowire axis) is positioned 16 nm away from its hemispherical termination. On the basis of a mode expansion analysis, the reflection coefficient r is calculated by normalizing the complex amplitude of the reflected field (A_{refl}) by that of the incident one (A_{inc}), $r = A_{\text{refl}}/A_{\text{inc}}$. The reflectance is then simply given by $|r|^2$, while the phase change upon reflection is $\operatorname{Arg} r$. The same definitions are also employed in the presence of an emitter (see below). To limit the computational cost in the case of an empty cavity, we exploit the cylindrical symmetry of the nanowire-dimer system by simulating only a quarter of it, imposing symmetric boundary conditions in both the y- and z-directions. Additionally, open boundaries in the x-direction are described by means of perfectly matched layers[15].

Note that the nanowire-nanorod and inter-nanorod gap sizes considered in this work (3 nm and 3 nm respectively) are sufficiently large to justify the neglect of non-local and quantum corrections to our purely classical electrodynamic modelling. For sub-nanometric junctions, fully quantum or semiclassical simulation methodologies may be required to ascertain the fundamental reflection and transmission coefficients of the nanowire guided mode under the influence of such effects as electron spill-out at metal-dielectric interfaces, non-local dynamical screening or quantum tunnelling across dielectric junctions. Nevertheless, as long as these coefficients can be obtained, the gCPA condition would still be established in the same manner.

Supplementary Note 6. Maxwell-Bloch simulations

To describe the interaction of a single quantum emitter with the plasmonic near-field of a waveguide-driven nanocavity in a self-consistent fashion, we couple the classical Maxwell's equations with the quantum mechanical optical Bloch equations[16,17]. Denoting the ground and excited states of the emitter by $|1\rangle$ and $|2\rangle$ respectively, the Hamiltonian describing its interaction with a classical external field is given by

$$\hat{H} = \hbar\omega_e\hat{\sigma}^\dagger\hat{\sigma} - \hat{\boldsymbol{\mu}}\cdot\mathbf{E} = \hbar\omega_e\hat{\sigma}^\dagger\hat{\sigma} - \boldsymbol{\mu}\cdot\mathbf{E}(\hat{\sigma}^\dagger + \hat{\sigma}),$$

where ω_e is the transition frequency, $\hat{\sigma} = |1\rangle\langle 2|$ and $\hat{\sigma}^\dagger = |2\rangle\langle 1|$ are the lowering and raising operators of the system respectively, and $\boldsymbol{\mu} = \langle 2|\hat{\boldsymbol{\mu}}|1\rangle$ is a transition dipole matrix element.

We regard the two-level emitter as an open quantum system which, in addition to its dipole interaction with an external field, experiences relaxation and dephasing. The temporal evolution of the emitter density operator under these conditions can be described by means of the following quantum master equation in Lindblad form[17],

$$\frac{\partial\hat{\rho}}{\partial t} = -\frac{i}{\hbar}[\hat{H},\hat{\rho}] + \frac{\gamma_\mathrm{r}}{2}(2\hat{\sigma}\hat{\rho}\hat{\sigma}^\dagger - \hat{\sigma}^\dagger\hat{\sigma}\hat{\rho} - \hat{\rho}\hat{\sigma}^\dagger\hat{\sigma}) + \frac{\gamma_\mathrm{p}}{2}(2\hat{\sigma}^\dagger\hat{\rho}\hat{\sigma} - \hat{\sigma}\hat{\sigma}^\dagger\hat{\rho} - \hat{\rho}\hat{\sigma}\hat{\sigma}^\dagger) + \frac{\gamma_\mathrm{d}}{2}(\hat{\sigma}_z\hat{\rho}\hat{\sigma}_z - \hat{\rho}), \tag{3}$$

where $\hat{\sigma}_z = |2\rangle\langle 2| - |1\rangle\langle 1|$, γ_r and γ_p are the incoherent relaxation and pumping rates respectively, and γ_d is the pure dephasing rate. Assuming the emitter to be localized to a single spatial grid cell of size Δx, we can write its time-dependent polarization $\mathbf{p}$ in terms of the corresponding macroscopic polarization density $\mathbf{P}$ featuring in Maxwell's equations via

$$\mathbf{p} = \Delta x^3\mathbf{P} = \mathrm{Tr}(\hat{\rho}\hat{\boldsymbol{\mu}}) = \boldsymbol{\mu}(\rho_{12} + \rho_{21}) = 2\boldsymbol{\mu}\mathrm{Re}(\rho_{12}). \tag{4}$$

Forming matrix elements of the left- and right-hand sides of Eq. (3) in a basis comprising the emitter ground and excited states, and using Eq. (4), we find the following system of coupled, partial differential equations for the density matrix elements ρ_{ij},

$$\frac{\partial^2\mathbf{p}}{\partial t^2} + 2\Gamma_\mathrm{e}\frac{\partial\mathbf{p}}{\partial t} + \left(\Gamma_\mathrm{e}^2 + \omega_\mathrm{e}^2\right)\mathbf{p} = -\frac{2\omega_\mathrm{e}}{\hbar}\boldsymbol{\mu}(\boldsymbol{\mu}\cdot\mathbf{E})(\rho_{22} - \rho_{11}) \tag{5}$$

$$\frac{\partial\rho_{22}}{\partial t} = -\frac{\partial\rho_{11}}{\partial t} = -\gamma\rho_{22} + \frac{1}{\hbar\omega_\mathrm{e}}\left(\frac{\partial\mathbf{p}}{\partial t} + \Gamma_\mathrm{e}\mathbf{p}\right)\cdot\mathbf{E}, \tag{6}$$

where $\gamma = \gamma_\mathrm{r} + \gamma_\mathrm{p}$ and $\Gamma_\mathrm{e} = \gamma_\mathrm{d} + \gamma/2$ is the total dephasing rate. For the calculation of the quantities γ and Γ_e, we disregard thermal pumping given its negligibility at room temperature, and set $\gamma_\mathrm{r} = 10^9$ rad/s (corresponding to a nanosecond-scale excited-state lifetime) as a typical order of magnitude for molecular and quantum dot emitters. Furthermore, we adopt $\gamma_\mathrm{d} = 4\times 10^{13}$ rad/s as a representative value for such single emitters at room temperature. As a result, $\Gamma_\mathrm{e} \approx \gamma_\mathrm{d}$. We also note that since $\gamma_\mathrm{p} \ll \gamma_\mathrm{r}$, the steady-state value of the excited-state population, $\rho_{22}^\mathrm{SS} = \gamma_\mathrm{p}/(\gamma_\mathrm{r} + \gamma_\mathrm{p}) \ll 1$ and so we have neglected ρ_{22}^SS in our derivation.

A well-known complication of such self-consistent numerical simulations is the need to carefully treat the divergent nature of the in-phase self-field of the point-like emitter, which can give rise to nonphysical frequency shifts and becomes particularly important for larger dipole moments[18,19]. To that end, we follow the self-interaction correction scheme proposed by Schelew *et al.*[19], for which more detailed analysis and discussion can be found in their work. Here, the total electric field entering Eqs. (5) and (6) is corrected by subtracting a numerically divergent contribution $\mathbf{E}^\mathrm{div}$, given by

$$\mathbf{E}^\mathrm{div} = -\frac{\mathbf{p}}{3\varepsilon_0\varepsilon_\mathrm{B}(\Delta x)^3}\left[1 + f(\Delta x)\right],$$

where ε_B is the dielectric constant of the embedding medium (here $\varepsilon_\mathrm{B} = 1$) and

$$f(\Delta x) = -\left(\frac{3}{4\pi}\right)^{2/3}\left(\frac{1.15\omega_\mathrm{e}\Delta x\sqrt{\varepsilon_\mathrm{B}}}{c}\right)^2.$$

In this work, we solve numerically the system comprising Maxwell's equations and the optical Bloch equations in a completely self-consistent fashion, where the local electric field (computed using the FDTD method) drives a polarization response from the quantum emitter (determined by solving the optical Bloch equations), which in turn couples back to the field.

Supplementary Note 7. cQED model of photoluminescence

To predict the photoluminescence (PL) spectrum of the free nanocavity-emitter system in Fig. 2a of the main text, we employ a simple theoretical model within the framework of cavity quantum electrodynamics (cQED). We assume that the lowest-order bonding dimer mode of the nanorods behaves as a single bosonic cavity mode with frequency ω_p and is characterized by the annihilation and creation operators $\hat{a}$ and $\hat{a}^\dagger$ respectively, while the emitter is treated as a quantum two-level system as described above. In the absence of dissipative effects, the coherent and conservative dynamics of the interacting cavity-emitter system is governed by the Jaynes-Cummings (JC) Hamiltonian which, under the unitary transformation $U = \exp\left[-i\omega_\mathrm{p} t(\hat{\sigma}^\dagger\hat{\sigma} + \hat{a}^\dagger\hat{a})\right]$, reads

$$\hat{H}_\mathrm{JC} = \hbar(\omega_\mathrm{e} - \omega_\mathrm{p})\hat{\sigma}^\dagger\hat{\sigma} + \hbar g(\hat{a}\hat{\sigma}^\dagger + \hat{a}^\dagger\hat{\sigma}).$$

The coupling strength is given by $g = \sqrt{F_{m=1}(\mathbf{r}_\mathrm{e},\omega_\mathrm{p})\gamma_0\kappa/2}$, where $F_{m=1}(\mathbf{r}_\mathrm{e},\omega_\mathrm{p})$ is the local Purcell factor of the lowest-order bonding mode at the position of the emitter $\mathbf{r}_\mathrm{e}$, γ_0 is the free-space decay rate, and κ is the cavity mode decay rate. Physically, the PL spectrum can be determined from the steady-state number of photons $\langle\hat{a}^\dagger\hat{a}\rangle$ in the system as a function of frequency, subject to pumping, dissipation and decoherence. To capture the impact of these effects, we describe the system dynamics via a quantum master equation for the density operator $\hat{\rho}$, $\partial\hat{\rho}/\partial t = \mathscr{L}\hat{\rho}$, where

$$\begin{aligned}\mathscr{L}\hat{\rho} = &-\frac{i}{\hbar}[\hat{H}_\mathrm{JC},\hat{\rho}] + \frac{\kappa}{2}(2\hat{a}\hat{\rho}\hat{a}^\dagger - \hat{a}^\dagger\hat{a}\hat{\rho} - \hat{\rho}\hat{a}^\dagger\hat{a}) + \frac{\gamma_\mathrm{nr}}{2}(2\hat{\sigma}\hat{\rho}\hat{\sigma}^\dagger - \hat{\sigma}^\dagger\hat{\sigma}\hat{\rho} - \hat{\rho}\hat{\sigma}^\dagger\hat{\sigma}) \\ &+ \frac{\Lambda}{2}(2\hat{\sigma}^\dagger\hat{\rho}\hat{\sigma} - \hat{\sigma}\hat{\sigma}^\dagger\hat{\rho} - \hat{\rho}\hat{\sigma}\hat{\sigma}^\dagger) + \frac{\gamma_\mathrm{d}}{2}(\hat{\sigma}_z\hat{\rho}\hat{\sigma}_z - \hat{\rho}).\end{aligned}$$

Here, $\gamma_\mathrm{d} = 26$ meV is the pure dephasing rate of the emitter as above, $\Lambda = 1$ meV is the incoherent pumping rate (chosen sufficiently small to render the system in the weak excitation regime) and γ_nr is a relaxation rate arising from higher-order, non-radiative plasmonic modes of the cavity, which are assumed to behave collectively as a single pseudomode[20]. We calculate γ_nr via $\gamma_\mathrm{nr}(\mathbf{r}_\mathrm{e},\omega) = F_{m>1}(\mathbf{r}_\mathrm{e},\omega)\gamma_0(\omega)$, where the Purcell factor due to the higher-order modes, $F_{m>1}(\mathbf{r}_\mathrm{e},\omega)$, is estimated by subtracting $F_{m=1}(\mathbf{r}_\mathrm{e},\omega)$ from the total Purcell factor $F_\mathrm{tot}(\mathbf{r}_\mathrm{e},\omega)$. The latter is rigorously defined by

$$F_\mathrm{tot}(\mathbf{r}_\mathrm{e},\omega) = \frac{\mathrm{Im}\left[\mathbf{n}_\mathrm{e}\cdot\mathbf{G}(\mathbf{r}_\mathrm{e},\mathbf{r}_\mathrm{e},\omega)\cdot\mathbf{n}_\mathrm{e}\right]}{\mathrm{Im}\left[\mathbf{n}_\mathrm{e}\cdot\mathbf{G}_0(\mathbf{r}_\mathrm{e},\mathbf{r}_\mathrm{e},\omega)\cdot\mathbf{n}_\mathrm{e}\right]},$$

where $\mathbf{G}(\mathbf{r}_\mathrm{e},\mathbf{r}_\mathrm{e},\omega)$ and $\mathbf{G}_0(\mathbf{r}_\mathrm{e},\mathbf{r}_\mathrm{e},\omega)$ are dyadic Green's functions pertaining to the nanorod dimer and free-space electromagnetic environments respectively[21]. In practice, $F_\mathrm{tot}(\mathbf{r}_\mathrm{e},\omega)$ can be calculated by monitoring the power radiated by an intracavity dipole source with the same position $\mathbf{r}_\mathrm{e}$, dipole moment magnitude $\mu = |\boldsymbol{\mu}|$ and orientation $\mathbf{n}_\mathrm{e}$, as well as frequency ω_e as the quantum emitter, via the standard relation

$$\frac{dW}{dt} = \frac{\omega^3\mu^2}{2\varepsilon\varepsilon_0 c^2}\mathrm{Im}\left[\mathbf{n}_\mathrm{e}\cdot\mathbf{G}(\mathbf{r}_\mathrm{e},\mathbf{r}_\mathrm{e},\omega)\cdot\mathbf{n}_\mathrm{e}\right],$$

in which ε is the relative permittivity of the material environment. Supplementary Figure 4 presents the multimode Purcell factors of the nanorod dimer cavity considered in Fig. 2a of the main text. The wavelength-dependence of the quantities $F_{m=1}$ and $F_{m>1}$ are described by means of multi-Lorentzian fits to the calculated F_tot, guided by its decomposition into radiative and non-radiative contributions. The decay rate of the bonding dimer mode can also be estimated from the full-width at half-maximum of the corresponding peak in F_tot, for which we obtain $\kappa = 0.126$ eV.

Finally, the PL lineshape can be determined by invoking the Wiener-Khintchine and quantum regression theorems in a similar spirit to previous works[22,23], ultimately yielding

$$S_\mathrm{PL}(\omega) \propto -\mathrm{Re}\left(\mathrm{Tr}\left\{\hat{a}^\dagger\left[\mathscr{L} - i(\omega-\omega_\mathrm{p})\hat{\mathscr{I}}\right]^{-1}\hat{a}\hat{\rho}_0\right\}\right), \tag{7}$$

where $\hat{\mathscr{I}}$ is the identity operator and $\hat{\rho}_0$ is the steady-state solution of the quantum master equation, satisfying $\mathscr{L}\hat{\rho}_0 = 0$. To utilize this result in practice, we express all operators in matrix form, adopting a basis formed by products of the cavity-mode Fock states and two-level system ground and excited states. Note that by virtue of the weak excitation regime characterized by $\langle\hat{a}^\dagger\hat{a}\rangle \ll 1$, we can restrict the calculation of the trace in Eq. (7) to only the zero- and one-photon cavity states.

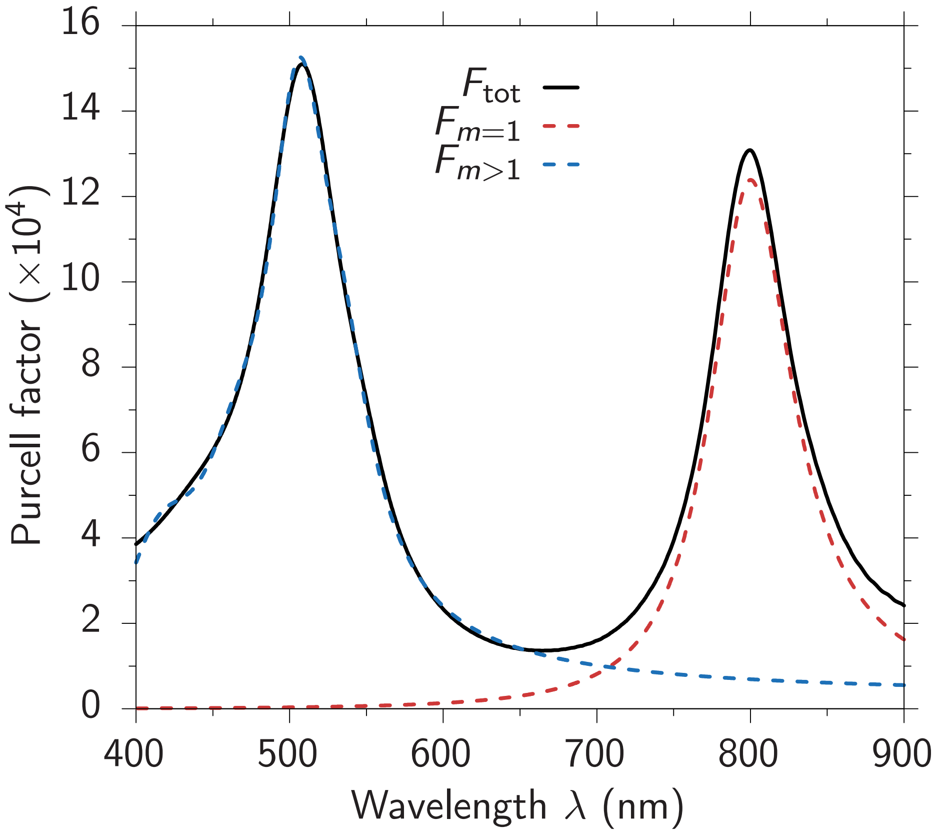

Supplementary Figure 4. Multimode Purcell factors of a nanorod dimer cavity. The structure comprises a pair of coaxial gold nanorods, each of length 114 nm and with an end-to-end separation of 3 nm. The total Purcell factor F_{tot} (solid black line) is decomposed according to the Purcell factor of the lowest-order bonding dimer mode, $F_{m=1}$ (red dashed line), and the Purcell factor arising from higher-order, non-radiative modes, $F_{m>1}$ (dashed blue line), described by means of multi-Lorentzian fits. Note that these quantities are calculated using a dipole source positioned at the center of the gap and oriented along the x-axis.

Supplementary Note 8. Quantum steady-state analysis of a continuously pumped Jaynes-Cummings system

In this section, we examine the steady-state dynamics of a JC system interacting with a weak, continuous-wave (CW) driving field that is near-resonant with one of the lowest polaritonic transitions (i.e., from the ground state to either of the first-rung polaritons in the JC ladder). As we show in the following, the use of a CW field necessarily induces semiclassical Rabi oscillations between the ground and excited states of the system, in stark contrast to our proposed quantum nanoplasmonic coherent perfect absorption (qnCPA) scheme that achieves truly stationary (time-independent) polariton populations.

Perturbation theory for steady states

Our analysis of a continuously pumped JC system is predicated on the quantum theory of steady states, first presented in a rigorous and unified manner by Sambe[24]. In particular, we shall rely on the perturbation-theoretic approach introduced therein, whose pertinent elements are briefly reviewed here before the technique is applied to the driven JC problem.

We consider a system with a time-independent Hamiltonian $H^{(0)}$ subject to a time-periodic interaction $V(t)$ with controllable strength. The total Hamiltonian $H(t,\lambda)$ is given by

$$H(t,\lambda) = H^{(0)} + \lambda V(t).$$

We shall assume that $V(t)$ has period $\tau = 2\pi/\omega$ [i.e., $H(t+\tau,\lambda) = H(t,\lambda)$] and that $\lambda \ll 1$. Following the general theory of Sambe[24], we define the steady states $|s(t,\lambda)\rangle$ and quasi-energies $\mathscr{E}(\lambda)$ as solutions of the eigenvalue problem

$$\left[\mathscr{H}^{(0)} + \lambda V(t)\right] |s(t,\lambda)\rangle = \mathscr{E}(\lambda)|s(t,\lambda)\rangle, \tag{8}$$

where $\mathscr{H}^{(0)} = H^{(0)} - i\hbar\partial/\partial t$. The operator featuring on the left-hand side of the above equation is linear and hermitian; in the quantum theory of steady states, it plays a role entirely analogous to that of the time-independent Hamiltonian for stationary states. The quasi-energies are real whilst the steady states exhibit the same periodicity as the interaction potential, $|s(t+\tau,\lambda)\rangle = |s(t,\lambda)\rangle$. Once they are known, the complete state vector of the system at time t can be expressed in the form $|\psi(t,\lambda)\rangle = e^{-i\mathscr{E}(\lambda)t/\hbar}|s(t,\lambda)\rangle$.

It should be noted that if $\{\mathscr{E}(\lambda), |s(t,\lambda)\rangle\}$ constitutes a solution of the steady-state eigenvalue problem in Eq. (8), then

$$\mathscr{E}'(\lambda) = \mathscr{E}(\lambda) + q\hbar\omega, \qquad |s'(t,\lambda)\rangle = e^{iq\omega t}|s(t,\lambda)\rangle \tag{9}$$

is also a solution for any integer q. However, the corresponding full state vectors are identical,

$$e^{-i\mathscr{E}t/\hbar}|s(t,\lambda)\rangle = e^{-i\mathscr{E}'t/\hbar}|s'(t,\lambda)\rangle.$$

As such, the set of solutions given by Eq. (9) specify physically equivalent steady states. Of course, this redundancy can be addressed by reducing any given quasi-energy $\mathscr{E}$ to a pre-defined interval or zone,

$$E - \frac{1}{2}\hbar\omega < \mathscr{E} \leq E + \frac{1}{2}\hbar\omega,$$

according to an arbitrary choice of real number E. In this way, physically distinct steady states can be characterized by their reduced quasi-energies, which all lie in the same zone.

Assuming a weak applied field, we seek a perturbative solution of Eq. (8) for the steady states and quasi-energies. To this end, we proceed as in standard Rayleigh-Schrödinger perturbation theory for stationary states, developing series expansions in powers of λ,

$$\mathscr{E}(\lambda) = \mathscr{E}^{(0)} + \lambda\mathscr{E}^{(1)} + \lambda^2\mathscr{E}^{(2)} + \dots \tag{10}$$

$$|s(t,\lambda)\rangle = |s^{(0)}(t)\rangle + \lambda|s^{(1)}(t)\rangle + \lambda^2|s^{(2)}(t)\rangle + \dots, \tag{11}$$

where $|s^{(n)}(t+\tau)\rangle = |s^{(n)}(t)\rangle$ for all n. The zeroth-order solutions $\{\mathscr{E}^{(0)}, |s^{(0)}(t)\rangle\}$ are obtained by solving the unperturbed eigenvalue problem

$$\mathscr{H}^{(0)}|s(t,0)\rangle = \mathscr{E}(0)|s(t,0)\rangle, \tag{12}$$

identifying $\mathscr{E}^{(0)} = \mathscr{E}(0)$ and $|s^{(0)}(t)\rangle = |s(t,0)\rangle$. They are readily determined via diagonalization of the time-independent Hamiltonian $H^{(0)}$; indeed, denoting the eigenvalues and eigenstates of $H^{(0)}$ by E_n and $|\phi_n\rangle$ respectively, the steady states and quasi-energies of the unperturbed system are

$$\mathscr{E}(0) = E_n + q\hbar\omega, \quad |s(t,0)\rangle = e^{iq\omega t}|\phi_n\rangle,$$

where a choice of zone as discussed above fixes q uniquely. Having solved the unperturbed eigenvalue problem in Eq. (12), the expansions in Eqs. (10) and (11) allow systematic perturbative corrections to be made to the unperturbed quasi-energies and steady states at ever higher orders in λ.

For the specific problem of interest here however, namely that of a quantum transition between two states induced by a near-resonant field, some care should be exercised. We consider once more the unperturbed eigenvalue problem of Eq. (12) and suppose that $H^{(0)}$ has two particular eigenvalues, say E_1 and E_2, which satisfy $E_2 = E_1 + \hbar\omega$. Then, it is readily seen that $|\phi_1\rangle$ and $e^{-i\omega t}|\phi_2\rangle$ are two eigenkets of $\mathscr{H}^{(0)}$ corresponding to distinct physical states of the system, but with the same eigenvalue E_1. Thus, even if the eigenvalue E_1 of $H^{(0)}$ can be treated as non-degenerate in stationary-state perturbation theory, it is degenerate as an eigenvalue of $\mathscr{H}^{(0)}$. This necessitates a degenerate formulation of perturbation theory for steady states, also presented in Ref.[24] and briefly summarised below.

Suppose that $\mathscr{E}_1^{(0)}$ and $\mathscr{E}_2^{(0)}$ are two distinct eigenvalues of $\mathscr{H}^{(0)}$ and that $|s_1^{(0)}(t)\rangle$ and $|s_2^{(0)}(t)\rangle$ are the corresponding steady states. Provided that the former are sufficiently close so as to satisfy $|(\mathscr{E}_1^{(0)} - \mathscr{E}_2^{(0)})/\lambda| \leq |\langle s_1^{(0)}|V(t)|s_2^{(0)}\rangle_{\mathrm{SS}}|$, the first-order approximate solution

$$\mathscr{E}(\lambda) = \frac{1}{2}\left(\mathscr{E}_1^{(0)} + \mathscr{E}_2^{(0)}\right) + \lambda\mathscr{E}^{(1)} + \mathscr{O}(\lambda^2)$$
$$|s(t,\lambda)\rangle = c_1|s_1^{(0)}(t)\rangle + c_2|s_2^{(0)}(t)\rangle + \mathscr{O}(\lambda),$$

can be found by obtaining non-trivial solutions of the secular equation

$$\begin{pmatrix} V_{11} + \Delta - \mathscr{E}^{(1)} & V_{12} \\ V_{21} & V_{22} - \Delta - \mathscr{E}^{(1)} \end{pmatrix}\begin{pmatrix} c_1 \\ c_2 \end{pmatrix} = \mathbf{0}, \tag{13}$$

where $\Delta = (\mathscr{E}_1^{(0)} - \mathscr{E}_2^{(0)})/2\lambda$ and $V_{mn} = \langle s_m^{(0)}|V(t)|s_n^{(0)}\rangle_{\mathrm{SS}}$. The subscript SS appearing in the above matrix elements denotes an inner product that pertains to the Hilbert space of the steady states of the system and whose definition is given in Ref.[24]. We leverage this formalism in the next section to explore the dynamics of a JC system subject to a weakly perturbing, near-resonant, CW field.

Application to the driven Jaynes-Cummings system

We now study the case in which $H^{(0)}$ is the Hamiltonian for the free JC system,

$$H^{(0)} = \hbar\omega_0\sigma^\dagger\sigma + \hbar\omega_0 a^\dagger a + \hbar g(a^\dagger\sigma + a\sigma^\dagger), \tag{14}$$

and $V(t)$ describes its interaction with a classical, external, CW driving field,

$$V(t) = Fe^{i\omega t} + F^\dagger e^{-i\omega t},$$

in which F may be proportional to either σ or a (i.e., the external field may couple either to the two-level emitter or the cavity mode).

The unperturbed steady states and quasi-energies [i.e., the solutions of Eq. (12)] are determined by the eigenstates and eigenvalues of the JC Hamiltonian in Eq. (14). These are precisely the well-known dressed or polariton states and corresponding energy levels of the JC ladder, which can be found in standard texts on quantum optics (for example, see Ref.[25]). In our present analysis of the CW-driven JC system, we focus on the ground state, $|\phi_1\rangle = |0\rangle \otimes |g\rangle$, and a polariton state at the first rung of the JC ladder, say the lower one $|\phi_2\rangle = (|0\rangle \otimes |e\rangle - |1\rangle \otimes |g\rangle)/\sqrt{2} = |l\rangle$, denoting the corresponding eigenenergies by E_1 and E_2 respectively. Here, $|0\rangle$ and $|1\rangle$ are vacuum and single-photon Fock states of the cavity mode, while $|g\rangle$ and $|e\rangle$ are the ground and excited states of the emitter. We also assume that $\hbar\omega \approx E_2 - E_1$, corresponding to near-resonant driving by the applied field.

Reducing the quasi-energies to a single zone, we can write the zeroth-order solutions as

$$\mathscr{E}_1^{(0)} = E_1, \qquad |s_1^{(0)}\rangle = |\phi_1\rangle$$
$$\mathscr{E}_2^{(0)} = E_2 - \hbar\omega, \qquad |s_2^{(0)}\rangle = e^{-i\omega t}|\phi_2\rangle.$$

The eigenvalues of the matrix in Eq. (13) are then found to be

$$\mathscr{E}_\pm^{(1)} = \pm\left[\Delta^2 + |\langle\phi_1|F|\phi_2\rangle|^2\right]^{1/2}$$

with $\Delta = (E_1 - E_2 + \hbar\omega)/2\lambda$, while the corresponding eigenvectors $(c_{1\pm}, c_{2\pm})^T$ are determined (up to a phase factor) by the relations

$$R_\pm = \frac{c_{1\pm}}{c_{2\pm}} = \frac{\langle\phi_1|F|\phi_2\rangle}{\mathscr{E}_\pm^{(1)} - \Delta}, \qquad |c_{1\pm}|^2 + |c_{2\pm}|^2 = 1.$$

The first-order solutions are thus given by

$$|\psi_\pm(t)\rangle = \left[c_{1\pm}|s_1^{(0)}\rangle + c_{2\pm}|s_2^{(0)}\rangle\right] e^{-i\left[\mathscr{E}^{(0)} + \lambda\mathscr{E}_\pm^{(1)}\right]t/\hbar},$$

with $\mathscr{E}^{(0)} = \left[\mathscr{E}_1^{(0)} + \mathscr{E}_2^{(0)}\right]/2$.

We now suppose that the system is prepared in the state $|\phi_1\rangle = |0\rangle \otimes |g\rangle$ at time $t = 0$. Correct to first order, the state at time $t > 0$ is described by

$$\begin{aligned}|\psi(t)\rangle = {} & \frac{e^{-i\mathscr{E}^{(0)}t/\hbar}}{R_+ - R_-}\left[R_+ e^{-i\lambda\mathscr{E}_+^{(1)}t/\hbar} - R_- e^{-i\lambda\mathscr{E}_-^{(1)}t/\hbar}\right]|\phi_1\rangle \\ & + \frac{e^{-i\mathscr{E}^{(0)}t/\hbar}}{R_+ - R_-} e^{-i\omega t}\left[e^{-i\lambda\mathscr{E}_+^{(1)}t/\hbar} - e^{-i\lambda\mathscr{E}_-^{(1)}t/\hbar}\right]|\phi_2\rangle.\end{aligned}$$

The probability $N_2(t)$ of finding the system in the state $|\phi_2\rangle$ at time t is finally given by

$$\begin{aligned}N_2(t) &= \left|\frac{e^{-i\lambda\mathscr{E}_+^{(1)}t/\hbar} - e^{-i\lambda\mathscr{E}_-^{(1)}t/\hbar}}{R_+ - R_-}\right|^2 \\ &= \frac{\lambda^2|\langle\phi_1|F|\phi_2\rangle|^2}{\hbar^2}\left(\frac{\sin\Omega t}{\Omega}\right)^2,\end{aligned} \tag{15}$$

where $\hbar\Omega = \left[\frac{1}{4}(E_1 - E_2 + \hbar\omega)^2 + \lambda^2|\langle\phi_1|F|\phi_2\rangle|^2\right]^{1/2}$. The probability $N_1(t)$ to find the system in the state $|\phi_1\rangle$ at time t is readily shown to be $N_1(t) = 1 - N_2(t)$. Note that in the main text we refer to the ground-state $|\phi_1\rangle$ as $|0\rangle$, and the polariton state $|\phi_2\rangle$ as $|j\rangle$ with $j = l, u$. Equation (15) is precisely the semiclassical Rabi formula; our analysis thus establishes that a weak, CW driving field, which is near-resonant with one of the first-rung polaritonic transitions of the JC system, induces a temporal cycling of population (i.e., semiclassical Rabi oscillations) between the ground and excited states.

Supplementary Note 9. Movies of the nanocavity-emitter system under qnCPA

The movies are available online and display the time-harmonic evolution of the electric near-field components E_x and E_z at the critical frequencies for qnCPA, in both the upper polariton state (Supplementary Movies 1 and 2, respectively) and the lower polariton state (Supplementary Movies 3 and 4, respectively). The left-hand side in each visualization presents a global system perspective, from which we observe the resonant feeding of the nanocavity-emitter system by the right-propagating nanowire plasmon mode. Indeed, as discussed in the main text, the precisely optimized combination of rod lengths, nanogap dimensions and frequency (see the caption of Fig. 3 in the main text) renders the hybrid system in a novel kind of impedance matching, whereby the nanowire excitation is transferred coherently to the nanocavity-emitter device with a complete suppression of back-reflected signals, preparing the latter in a pure, plasmon-emitter dressed state with the utmost selectivity, as afforded by the frequency-specificity of the qnCPA condition itself.

In addition to the global system view, we also provide a zoom-in display of the time-harmonic near-field evolution in the nanocavity-emitter region (directly corresponding to the region demarcated by a black box in Fig. 4 of the main text). It is in this comparatively small region ($\sim$ 3 nm wide) that we can identify the key distinguishing spatial characteristics of the two polaritons, namely an in-phase relationship between the local nanocavity and emitter fields for the upper polariton state (Supplementary Movies 1 and 2) and an antiphase one for the lower polariton state (Supplementary Movies 3 and 4).

References

1. Chong, Y. D., Ge, L., Cao, H. & Stone, A. D. Coherent perfect absorbers: Time-reversed lasers. *Phys. Rev. Lett.* **105**, 053901 (2010).
2. Baranov, D. G., Krasnok, A., Shegai, T., Alù, A. & Chong, Y. Coherent perfect absorbers: linear control of light with light. *Nat. Rev. Mater.* **2**, 17064 (2017).
3. Haus, H. A. *Waves and Fields in Optoelectronics* (Prentice-Hall, Englewood Cliffs, 1984).
4. Grimm, P., Razinskas, G., Huang, J.-S. & Hecht, B. Driving plasmonic nanoantennas at perfect impedance matching using generalized coherent perfect absorption. *Nanophotonics* **10**, 1879–1887 (2021).
5. Sweeney, W. R., Hsu, C. W. & Stone, A. D. Theory of reflectionless scattering modes. *Phys. Rev. A* **102**, 063511 (2020).
6. Stone, A. D., Sweeney, W. R., Hsu, C. W., Wisal, K. & Wang, Z. Reflectionless excitation of arbitrary photonic structures: a general theory. *Nanophotonics* **10**, 343–360 (2021).
7. Dorfmüller, J. *et al.* Fabry-pérot resonances in one-dimensional plasmonic nanostructures. *Nano Lett.* **9**, 2372–2377 (2009).
8. Taminiau, T. H., Stefani, F. D. & van Hulst, N. F. Optical nanorod antennas modeled as cavities for dipolar emitters: Evolution of sub- and super-radiant modes. *Nano Lett.* **11**, 1020–1024 (2011).
9. Zhu, Z. & Brown, T. G. *Opt. Express* **10**, 853–864 (2002).
10. Olmon, R. L. *et al.* Optical dielectric function of gold. *Phys. Rev. B* **86**, 235147 (2012).
11. Krasnok, A. *et al.* Anomalies in light scattering. *Adv. Opt. Photon.* **11**, 892–951 (2019).
12. Bergman, D. J. & Stockman, M. I. Surface plasmon amplification by stimulated emission of radiation: Quantum generation of coherent surface plasmons in nanosystems. *Phys. Rev. Lett.* **90**, 027402 (2003).
13. Noh, H., Chong, Y., Stone, A. D. & Cao, H. Perfect coupling of light to surface plasmons by coherent absorption. *Phys. Rev. Lett.* **108**, 186805 (2012).
14. Azzam, S. I. *et al.* Ten years of spasers and plasmonic nanolasers. *Light. Sci. Appl.* **9**, 90 (2020).
15. Taflove, A. & Hagness, S. C. *Computational Electrodynamics: The Finite-Difference Time-Domain Method* (Artech House, Boston, 2005).
16. Boyd, R. W. *Nonlinear Optics* (Academic Press, Burlington, 2008).
17. Carmichael, H. J. *Statistical Methods in Quantum Optics 1* (Springer-Verlag, Berlin, 1999).
18. Deinega, A. & Seideman, T. Self-interaction-free approaches for self-consistent solution of the maxwell-liouville equations. *Phys. Rev. A* **89**, 022501 (2014).
19. Schelew, E., Ge, R.-C., Hughes, S., Pond, J. & Young, J. F. Self-consistent numerical modeling of radiatively damped lorentz oscillators. *Phys. Rev. A* **95**, 063853 (2017).
20. Delga, A., Feist, J., Bravo-Abad, J. & Garcia-Vidal, F. J. Quantum emitters near a metal nanoparticle: Strong coupling and quenching. *Phys. Rev. Lett.* **112**, 253601 (2014).
21. Novotny, L. & Hecht, B. *Principles of Nano-optics* (Cambridge University Press, 2012).
22. Laussy, F. P., del Valle, E. & Tejedor, C. Luminescence spectra of quantum dots in microcavities. i. bosons. *Phys. Rev. B* **79**, 235325 (2009).
23. del Valle, E., Laussy, F. P. & Tejedor, C. Luminescence spectra of quantum dots in microcavities. ii. fermions. *Phys. Rev. B* **79**, 235326 (2009).
24. Sambe, H. Steady states and quasienergies of a quantum-mechanical system in an oscillating field. *Phys. Rev. A* **7**, 2203–2213 (1973).
25. Gerry, C. C. & Knight, P. L. *Introductory Quantum Optics* (Cambridge University Press, 2005).
