# Supplementary material for: Room-temperature quantum nanoplasmonic coherent perfect absorption
Source: Nat Commun. 2024 Jul 27;15:6324. doi: 10.1038/s41467-024-50574-9 (PMC11282272; doi:10.1038/s41467-024-50574-9)
Supplement: Supplementary file 3 — Description of Additional Supplementary Files [file 41467_2024_50574_MOESM3_ESM.pdf]

Description of Additional Supplementary Files

File Name: Supplementary Movie 1

Description: Dynamic evolution of the *x*-electric near-field component of a waveguide-driven nanocavity-emitter system at the critical frequency for qnCPA in the upper polariton state. The global field distribution is displayed on the left-hand side, whilst a magnified view of the nanocavity-emitter region is shown on the right-hand side. See Supplementary Information, Supplementary Note 9 for a full description of the movies.

File Name: Supplementary Movie 2

Description: Dynamic evolution of the *z*-electric near-field component of a waveguide-driven nanocavity-emitter system at the critical frequency for qnCPA in the upper polariton state. The global field distribution is displayed on the left-hand side, whilst a magnified view of the nanocavity-emitter region is shown on the right-hand side. See Supplementary Information, Supplementary Note 9 for a full description of the movies.

File Name: Supplementary Movie 3

Description: Dynamic evolution of the *x*-electric near-field component of a waveguide-driven nanocavity-emitter system at the critical frequency for qnCPA in the lower polariton state. The global field distribution is displayed on the left-hand side, whilst a magnified view of the nanocavity-emitter region is shown on the right-hand side. See Supplementary Information, Supplementary Note 9 for a full description of the movies.

File Name: Supplementary Movie 4

Description: Dynamic evolution of the *z*-electric near-field component of a waveguide-driven nanocavity-emitter system at the critical frequency for qnCPA in the lower polariton state. The global field distribution is displayed on the left-hand side, whilst a magnified view of the nanocavity-emitter region is shown on the right-hand side. See Supplementary Information, Supplementary Note 9 for a full description of the movies.
